# Nutritional Composition and Antioxidant Properties of the Fruits of a Chinese Wild *Passiflora foetida*

**DOI:** 10.3390/molecules23020459

**Published:** 2018-02-19

**Authors:** Ya Song, Xiao-Qun Wei, Mei-Ying Li, Xue-Wu Duan, Yuan-Ming Sun, Rui-Li Yang, Xiang-Dong Su, Ri-Ming Huang, Hong Wang

**Affiliations:** 1 Guangdong Provincial Key Laboratory of Food Quality and Safety, College of Food Science, South China Agricultural University, Guangzhou 510642, China; songya_1990@163.com (Y.S.); weixqun@scau.edu.cn (X.-Q.W.); lmy1982@scau.edu.cn (M.-Y.L.); ymsun@scau.edu.cn (Y.-M.S.); rlyang77@scau.edu.cn (R.-L.Y.); 2 Guangdong Provincial Key Laboratory of Applied Botany, South China Botanical Garden, Chinese Academy of Sciences, Guangzhou 510650, China; xwduan@scbg.ac.cn; 3 Department of Pharmacy and Pharmacology, University of Bath, Bath BA2 7AY, UK; prsxs@bath.ac.uk

**Keywords:** *Passiflora foetida*, fruit, nutritional composition, antioxidant properties, UPLC-MS, phenolic compound

## Abstract

The aim of this work was to evaluate the main nutrients and their antioxidant properties of a Chinese wild edible fruit, *Passiflora foetida,* collected from the ecoregion of Hainan province, China. The analytical results revealed that *P. foetida* fruits were rich in amino acids (1097 mg/100 g in total), minerals (595.75 mg/100 g in total), and unsaturated fatty acids (74.18 g/100 g in total fat). The lyophilized powder of edible portion contained the higher polyphenols content than the inedible portion powder. The UPLC-Q-TOF-MS^E^ analysis of the extractable and non-extractable phenolics indicated the presence of 65 compounds including 39 free phenolics, 14 insoluble-glycoside-phenolics, and 22 insoluble-ester-phenolics. In addition, the non-extractable phenolics obtained by alkali hydrolysis showed significant antioxidant activities by/through DPPH and ABTS radical scavenging. These findings of *P. foetida* fruits, for the first time, suggest that these polyphenol-rich fruits may have potential nutraceutical efficacies.

## 1. Introduction 

In recent years, consumers have becime increasingly interested in health and diet, resulting in an increasing demand for robust and comprehensive nutritional data of food. The nutritional and chemical compositions of many edible agrestal fruits are of considerable interest to those wishing to understand the basis of the nutritional value and to gain benefits arising from consuming fruits. 

*Passiflora*, a genus of the family, Passifloraceae, known also as the passion flower or passion vine, is a genus of about 500 species of flowering plants, which possess important nutritional and medicinal properties [1]. The Passifloraceae family has a wide pantropical distribution. *Passiflora* itself is widely distributed in South America, East Asia, and New Guinea. Some species of *Passiflora* have been naturalized beyond their native ranges. For example, *P*. *caerulea* now grows wildly in Spain. *P*. *edulis* and its yellow relative, flavicarpa, have been introduced into many tropical regions as commercial crops. Additionally, *Passiflora* species also have a long history of applications in traditional medicine [1]. For example, they are used as analgesic agents, or for the treatment of insomnia, hysteria, and epilepsy [2]. Previous phytochemical investigations on *Passiflora* species have shown the presence of β-carboline harmala alkaloids, coumarins, maltol, phytosterols and cyanogenic glycosides [3]. *Passiflora* is also rich in phenolic compounds, amino acid α-alanine, and organic acids, including butyric, formic, oleic, linoleic, malic, myristic, linolenic, and palmitic acids [4]. Esters like ethyl butyrate, n-hexyl butyrate, ethyl caproate, and n-hexyl caproate give the unique flavor and appetizing smell [5]. The main saccharides in the fruit are d-glucose, d-fructose, and raffinose [6]. In terms of enzymes, *Passiflora* has been found to be rich in catalase, pectin methylesterase and β-galactosidase [7].

*P. foetida*, commonly named as wild maracuja, bush passionfruit, marya-marya, wild water lemon, stinking passionflower, love-in-a-mist or running pop, is a species of passion flowers, which is native to the Southwestern United States (Southern Texas and Arizona), the Caribbean, Mexico, Central America, and South America [8]. This plant is distributed in tropical regions around the world, such as Southeast Asia, South China, and Hawaii. It is a creeping vine, like other members of the genus *Passiflora*, and yields an edible fruit. The specific epithet, foetida, means “stinking” in Latin and refers to the strong aroma emitted by damaged foliage. Although a few phytochemical investigations on *P. foetida* have shown the absence of bioactive C-glycosylflavonoids, polyketides [3] flavonoids [9] and phenolic compounds [2], the pharmacological evaluation of *P. foetida* has revealed that the extracts of *P. foetida* possess antioxidant, analgesic, anti-inflammatory, antipyretic [2], antibacterial [10] and anti-osteoporotic activities [11]. Up until now, there has been no systematic evaluation of the nutritional composition of *P. foetida*, especially in regard to the investigation of extractable and non-extractable polyphenols, even though they have received much attention in recent years due to their different bioavailable metabolites following interaction with microbiota in the colon and effects on the human body’s health [12]. Therefore, the nutritional and chemical composition of *P. foetida* has stimulated considerable attention, to understand their potential influence on health.

The purpose of this study was to evaluate the nutritional components and antioxidant properties of *P. foetida* fruit obtained from Hainan province, China. This was the first time that the nutrient value of the edible fruits of *P. foetida* has been investigated, and an analysis of the extractable and non-extractable phenolics by using the rapid ultra-performance liquid chromatography-quadrupole time-of-flight mass spectrometry (UPLC-Q-TOF-MS^E^) has been completed. In addition, the antioxidant activities of the extractable and non-extractable phenolics were evaluated. Thus, these findings may give a better understanding of the nutritional roles, and additional health benefits of consuming *P. foetida* fruits.

## 2. Material and Methods

### 2.1. Plant Material

Fruits of *P. foetida* were collected from Gancheng town, Dongfang city, Hainan province, China (latitude 18 43′ 12′′ E, longitude 108 36′ 46′′ N), in September 2016. The specimen was identified by Professor Hongfeng Chen from South China Botanical Garden, Chinese Academy of Sciences. A voucher specimen (20160901) was deposited in the Guangdong Provincial Key Laboratory of Food Quality and Safety, China. The fruits were harvested manually, according to the ripening stage. All ripening stage samples were selected in agreement with those in which the fruits are usually consumed. After collection, the fruits were packed in a portable refrigerator (4 °C) until they were transported to the laboratory (2–3 h). Some of the samples were stored at −80 °C until proximate analysis. The other samples were manually divided into the edible portion (pulp and seeds) and non-edible portion (peel), which were carefully separated from the flesh using a sharp knife. Then, the samples were frozen in liquid nitrogen and stored at −80 °C until analysis. Some of the samples were lyophilized and smashed to obtain the powder which was then placed in oxygen barrier bags and stored at −80 °C until analysis.

### 2.2. Standards and Reagents

6-Hydroxy-2,5,7,8-tetramethylchroman-2-carboxylic acid (Trolox), 1,1-diphenyl-2-picryl-hydrazl (DPPH), and 2,2-azinobis-(3-ethylbenzthiazoline-6-sulfonic acid) (ABTS) were purchased from Aladdin Industrial Corporation (Shanghai, China). Folin–Ciocalteu reagent was purchased from Solarbio Life Science (Beijing, China). All reagents were of analytical grade. All sugars, organic acids, gallic acid and rutin standards were obtained from Aladdin Industrial Corporation (Shanghai, China).

### 2.3. Physicochemical Characteristic

Twenty-five ripe fruits were measured. Polar and equatorial lengths were measured by a vernier (LabQuest, Vernier, OR, USA). Skin color in intact fruit was determined by a chromameter (CR-300, Minolta, TYO, Japan) and expressed on the CIELAB scale (L * = lightness, a * = red-green hue, b * = yellow-blue hue). 

### 2.4. Nutritional Determinations

#### 2.4.1. Proximate Analysis 

Proximate analysis of the chemical composition (moisture, ash, crude protein and crude fat) of the *P. foetida* fruit was conducted in accordance with the Association of Official Analytical Chemists methods [13]. Moisture content was determined by heating the fresh sample at 105 °C overnight until a constant weight. Ash content was determined by weighing the residue obtained after incineration at 550 °C for 4 h until constant weight. Crude protein content (N × 4.38) was determined using the Kjeldahl method. Crude fat content was determined by Soxhlet extraction with petroleum ether as the solvent. The pH value was measured with a pH meter (FiveEasyPlus™, METTLER TOLEDO, Zurich, Switzerland). Total carbohydrate content was determined using the phenol–sulfuric acid method [14]. Total energy was calculated using the following equation [15]:

Total Energy (kJ) =17 × (g crude protein + g total carbohydrate) + 37 (g crude fat).(1)

#### 2.4.2. Sugars Analysis

A quantity of 0.01 g of frozen sample was rapidly powdered with liquid nitrogen in a mortar and then transferred to a centrifuge tube with 5 mL deionized water. The reaction mixture was placed in a water bath for 20 min at 35 °C and centrifuged at 4000 rpm for 10 min. The extraction was repeated three times. The supernatant was made up to a constant volume of 25 mL. A chromatographic separation of sugars involved acetonitrile: water (70:30, *v*/*v*) as the mobile phase at a flow rate of 0.8 mL/min with an Asahipak NH_2_P-50-4E (4.5 μm, 4.6 mm × 250 mm) column (Shodex, Tokyo, Japan). Eluted peaks were detected with a SHODEX RI101 refractive index detector (JASCO International Co., Ltd., Tokyo, Japan). Sugar concentrations were calculated by a standard curve method and expressed in grams (g) per 100 g fresh edible portion. 

#### 2.4.3. Organic Acids Analysis

An 0.5 g frozen sample was rapidly powdered with liquid nitrogen in a mortar and then transferred to a centrifuge tube with 15 mL of 50 mM KH_2_PO_4_ solution (adjusted to pH 2.9 with phosphoric acid). The reaction mixture was placed in a water bath for 60 min at 75 °C and centrifuged at 4000 rpm for 15 min. The extraction was repeated three times. The supernatant was made up to a constant volume of 50 mL. Then, the mixture of the supernatant was collected for high-performance liquid chromatography (HPLC) analysis. The chromatographic conditions for organic acids were 50 mM KH_2_PO_4_ solution, with a flow rate of 0.8 mL/min. A C_18_ column (5 μm, 4.6 × 250 mm) (Shimadzu, Tokyo, Japan), with an ultraviolet detector (SPD-20A, Shimadzu, Tokyo, Japan) was used, with a detection wavelength of 215 nm. Organic acid concentrations were calculated by a standard curve method and expressed in milligrams (mg) per 100 g fresh edible portion. 

#### 2.4.4. Amino Acids Analysis

The amino acid contents were detected by ion-exchange chromatography using an amino acid Analyzer (L-8800, Hitachi, Tokyo, Japan) following acid hydrolysis with HCl (6 M) in sealed ampoules in an oven (Bio technics, Mumbai, India) at 110 °C for 22 h. Excess acid was removed by continuous flash evaporation under reduced pressure (Buchi, Flawil, Switzerland) and the sample was then dissolved in citrate buffer (pH 2.2) [16]. An aliquot (20 μL) of the sample was loaded into the automated amino acid analyzer. The quantification of each amino acid was based on a standard chromatogram, derived from standard amino acids and expressed in milligrams (mg) per 100 g fresh edible portion. 

#### 2.4.5. Minerals Analysis 

A dried fruit sample (0.5 g) was digested in 2 mL concentrated HNO_3_ in a microwave oven (MILLSTONE, Cremona, Italy) and then diluted with distilled water to 25 mL. This solution was filtered before storage. A blank digest was carried out in the same way [17]. Minerals and elements were measured by Inductively Coupled Plasma-Optical Emission Spectrometry (ICP-OES) (DV5300, PerkinElmer, Boston, MA, USA) method for calcium, iron, potassium, magnesium, sodium and phosphorous and the Inductively Coupled Plasma-Mass Spectrometry (ICP-MS) (ELAN DRC-e, PerkinElmer, Boston, MA, USA) method for copper, manganese and zinc. The results are expressed in milligrams (mg) per100 g fresh edible portion.

#### 2.4.6. Fatty Acids Analysis

The fatty acid profile was obtained by a gas chromatography analysis after esterification of the total lipids. The extracted lipids were saponified with 1 M KOH/MeOH at 60 °C for 20 min. Then, the released fatty acids were esterified by using 2 M HCl/MeOH at 60 °C for 20 min. An HPLC-grade hexane solvent was used to extract the methylated fatty acids. After that, the analysis was performed by using a GC trace gas chromatograph (7890B-5977A, Agilent, Palo Alto, CA, USA) equipped with a flame ionisation detector. For the chromatographic separation, an HP-5 MS capillary column (0.25 mm in diameter, 30 m in length) coated with a polar stationary phase of 0.2 μm thickness (Agilent, Palo Alto, CA, USA) was used. The carrier gas, hydrogen, was maintained at a constant pressure of 200 kPa. The column temperature was programmed as follows: 50 °C for 1 min; 50–200 °C for 5 min (5 °C/min); 220 °C for 10 min. The detector temperature was set at 230 °C. Hydrogen and air flow rates for the detector were maintained throughout all runs at 35 and 350 mL/min, respectively. A calibration mixture of fatty acid standards was processed in parallel. The data were analyzed by the Chromquest 3.0 software (Thermo Fisher Scientific, Waltham, MA, USA).

### 2.5. Extraction of Phenolic Compounds 

#### 2.5.1. Extractable Phenolics (EP)

About 4 g of each sample was transferred to a tube with 20 mL methanol/water (50:50 *v*/*v*) solution. The tubes were vortexed for 3 min, then extracted for 1 h in an ultrasonic water bath at room temperature. The tubes were then centrifuged at 4000 rpm for 10 min and the supernatants were collected. And 20 mL acetone/water (70:30 *v*/*v*) was added to tubes, then all treatments (vortexing, extraction, and centrifugation) were repeated. Two extracts were combined and transferred into tubes, then directly used for the determination of phytochemicals and antioxidant capacity [18]. 

#### 2.5.2. Non-Extractable Phenolics (NEP)

The NEPs were obtained by hydrolyzing after adding acid or alkali by the method described in the literature [12,18,19]. The residue, left after the extraction that was described previously, was moved into a fuming cupboard for 2 h, and lyophilized until dry. About 0.5 g residue was transferred to a tube with 20 mL methanol and 2 mL concentrated sulphuric acid (18 M). Samples were agitated for 1 min and shaken in a water bath at 85 °C for 10 h; samples were then centrifuged (4000 rpm for 10 min), and the supernatant was collected. After washing with a minimal volume of distilled water and re-centrifuging, the final volume was taken up to 40 mL. 

A quantity of 0.1 g of residue was placed in a tube and mixed with 2 mL NaOH (4 M). Furthermore, the samples were incubated in a temperature-controlled ultrasonic water bath (60 °C, for 45 min). After hydrolysis, the samples were neutralized by HCl (12 M). The liberated NEP was then extracted using 4 mL MeOH (containing 0.1% formic acid) followed by vortexing for 2 min. The tubes were centrifuged at 4000 rpm for 10 min at 4 °C. Extraction was performed twice and the final volume was adjusted to 20 mL using 100% methanol.

### 2.6. Measurements of Antioxidant Capacity and Functional Phytochemicals

#### 2.6.1. Extraction and Quantification of Total Phenolic Contents (TPC) and Total Flavonoid Contents (TFC)

The TPC was determined by using the Folin–Ciocalteu reagent. Briefly, appropriate dilutions of extracts (1 mL) were oxidized with 0.5 mL Folin–Ciocalteu reagent (0.4 M), and the reaction was neutralized with NaCO_3_ (7%, *w*/*v*). The absorbance of the resulting blue color was measured at 760 nm against an appropriate blank after 2 h of reaction at room temperature [17,20]. Gallic acid was used as the standard, and the data were expressed as milligram (mg) gallic equivalents (GAE) per 100 g lyophilized powder.

Total flavonoid content (TFC) was measured using a modified colorimetric method [21]. To measure total flavonoid content, the reaction system consisted of 1 mL of sample and 0.3 mL NaNO_2_ (5%, *w*/*v*). After reacting for 6 min, 0.3 mL Al(NO_3_)_3_ (10%, *w*/*v*) was added and left to stand for 6 min; Then, 2.0 mL NaOH (4%, *w*/*v*) was added and ethanol was supplemented to give a total volume of 10 mL. The absorbance was measured at 510 nm after reacting for 15 min. Rutin was used as the standard, and the data were expressed as milligram (mg) rutin equivalents (RUE) per 100 g lyophilized powder.

#### 2.6.2. Antioxidant Activity by DPPH and ABTS

For the DPPH radical scavenging assay, 1 mL extract was mixed with 2mL DPPH into ethanol (150 μg/mL) [22]. After being kept in the dark for 15 min, the absorbance of the solution was measured at 519 nm in a visible spectrophotometer (Labomed Inc, 23 RS, Fremont, CA, USA). Appropriate blanks (ethanol) and standards (Trolox solutions in ethanol) were detected simultaneously. The percentage of scavenging ratios and SC_50_ values were calculated, and the lower absorbance indicated a higher free radical scavenging activity. 

ABTS (7.4 mM) radical cation (ABTS·^+^) solution was produced through a reaction of ABTS with 2.6 mM potassium persulfate persulphate (K_2_S_2_O_8_), and the mixture was placed in the dark at room temperature for 12 h before use. The ABTS radical was diluted using K_2_S_2_O_8_ to give an absorbance of about 0.700 ± 0.020 at 734 nm. To measure antioxidant capacity, 0.4 mL extract was mixed with 1.6 mL radical solution. Absorbance was monitored at 734 nm for 6 min. The percentages of scavenging ratios and SC_50_ values were calculated [22]. Appropriate blanks (ethanol) and standards (Trolox solutions in ethanol) were detected simultaneously.

#### 2.6.3. Chromatography and Mass Spectrometry

After being filtered through an 0.22 μm syringe filter, 2 μL of the supernatant was injected into the UPLC-Q-TOF-MS^E^ system for analysis. UPLC-Q-TOF-MS^E^ was performed in an Agilent 1290/maXis impact UPLC™ system (Bruker Corporation, Karlsruhe, Germany). Chromatographic separation was carried out at 30 °C in a ZORBAX RRHD UPLC SB-C_18_ column (2.1 × 50 mm, 1.8 μm) with mobile phases A (0.1% formic acid) and B (methanol). The flow rate was set at 200 μL/min. The gradient profile was as follows: 0–4 min, 10% B; 4–11 min, 10–85% B; 11–14 min, 85% B; 14–17 min, 85–10% B; 17–20 min,10% B. The mass acquisition range was from *m*/*z* 50 to 1500, and each sample was analyzed in negative mode. The MS source temperature was 180 °C, while the desolvation temperature was 450 °C and the desolvation gas flow was 4.0 L/min. The capillary and cone voltages were 3.5 kV and 2 kV, respectively. 

### 2.7. Statistical Analysis

All experiments were carried out in triplicate, and the results were expressed as mean values based on fresh matter. The extractions were performed in triplicate, and each sample was quantified in duplicate. Means were compared using Tukey’s honestly significant difference (HSD) multiple comparison test by SPSS19.0 software (IBM Corporation, Armonk, NY, USA).

## 3. Results and Discussion 

### 3.1. Physicochemical Characteristics of the Edible Portion

The appearance description of *P. foetida* fruits are presented in Table 1 and proximate compositions are shown in Table 2. The fruits are relatively small in the Passifloraceae family, compared to *P. quadrangularis* [23]. The skin of fruits is yellow and sleek, wrapped in a layer of a division of filamentous bracts. The edible portion ranged from 62.73% to 89.85%, and the fruits were consumed with sweet and sour pulp and juice, as well as black and small seeds.

### 3.2. Nutritional Composition

#### 3.2.1. Sugars and Organic Acids

Four soluble sugars, namely sucrose, fructose, glucose, and maltose, were identified, and their contents were assessed in this study (Table 3). The most abundant sugars in *P. foetida* fruits were found to be glucose (47.90%) and fructose (47.90%), while sucrose was found only in small amounts (4.20%) and maltose was inexistent. Our results disagree with the results of other passionfruits [24], in which sucrose was the most abundant sugar (about 60%); the rest were glucose and fructose (about 40%).

The five organic acids in *P. foetida* fruits are oxalic acid, tartaric acid, malic acid, ascorbic acid and citric acid, and their contents are shown in Table 3. Oxalic acid (29.17%) and citric acid (50.00%) are the major organic acids of the fruit among all organic acids, and the analysis showed that they account for 79.17% of the total acid content. Citric acid was also the most abundant organic acid in the passionfruit samples, while other organic acids were of very low concentrations [24].

#### 3.2.2. Amino Acids 

*P. foetida* fruits are relatively poor sources of dietary protein, and the free amino acid compositions of the fruit in this study (17 amino acids were detected) are shown in Table 3. In the study, the glutamic acid/glutamate (203 mg per 100 g of the edible part) and arginine (140 mg per 100 g of the edible part) contents were the highest among the 17 detected amino acids, and essential amino acid (EAA) content accounted for 26.07% of the total amino acid content. Meanwhile, the contents of histidine and some essential amino acids, such as threonine, isoleucine, leucine, lysine, and valine were higher than the requirements in human nutrition [25]. Among the amino acids, glutamate is responsible for palatable taste and glycine is responsible for sweet taste [15]. The percentages (18.51%, 5.74% and 10.02%, respectively) of savory amino acids (glutamic acid, glycine and aspartic acid) accounted for more than one-third of the total amino acids in the fruits.

#### 3.2.3. Minerals

The analysis of minerals in *P. foetida* fruits indicated that potassium (451 mg per 100 g of edible part) was the most abundant among the macroelements, followed by phosphorus (86 mg per 100 g of the edible part) and magnesium (40 mg per 100 g of the edible part). The potassium and magnesium contents were higher than those of other *Passiflora* fruits, such as *P. edulis* Sims (348 and 29 mg per 100 g of the edible part, respectively), *P. edulis* f. *flavicarpa* Deg (278 and 17 mg per 100 g of the edible part, respectively), and *P. alata* Curtis (375.42 and 19.82 mg per 100 g of the edible part, respectively), but the contents of sodium and calcium (9.57 and 6.27 mg per 100 g of the edible part, respectively) were similar to those of other *Passiflora* fruits (6–28 and 4–12 mg per 100g of the edible part, respectively) [26]. Furthermore, *P. foetida* fruits had a higher content of phosphorus (86 mg per 100 g of edible part) than that of other *Passiflora* fruits, such as *P. edulis* Sims (38 mg per 100 g of the edible part), *P. edulis* f. *flavicarpa* Deg (13 mg per 100 g of the edible part), *P. alata* Curtis (34.95 mg per 100 g of the edible part), *P. ligularis* Juss (64 mg per 100 g of the edible part) and *P. mollissima* L.H. Bailey (18 mg per 100 g of the edible part) [27].

Regarding the microelements, the quantification results of the fruits indicated that the fruits contained a large amount of zinc (1.03 mg per 100 g of the edible part), followed by iron (0.89 mg per 100 g of the edible part), manganese (0.32 mg per 100 g of the edible part), and copper (0.21 mg per 100 g of the edible part). According to the literature, the contents of zinc and copper were higher than those of *P. edulis* Sims (0.1 and 0.086 mg per 100 g of the edible part, respectively) and *P. edulis* f. *flavicarpa* Deg (0.05 and 0.053 mg per 100 g of the edible part, respectively). The iron content was semblable as *P. ligularis* Juss (0.9 mg per 100 g of the edible part), and a little bit lower than that of *P. edulis* Sims and *P. alata* Curtis (1.6 and 1.06 mg per 100 g of the edible part, respectively), but much higher than *P. edulis* f. *flavicarpa* Deg and *P. mollissima* L.H. Bailey (0.24 and 0.4 mg per 100 g of the edible part, respectively) [26]. Moreover, manganese is an inorganic nutrient involved in many important enzymes and/or proteins and thereby, in many physiological functions of the organism [28]. The manganese content (0.32 mg per 100 g of the edible part) was not only higher than that of other *Passiflora* fruits, such as *P. edulis* and *P. ligularis* (0.12 and 0.18 mg per 100 g of the edible part, respectively), but also higher than that of other tropical fruits, such as papaya, jujuba, *Sechium edule* and *Ananas comosus* (0.03, 0.13, 0.07 and 0.26 mg per 100 g of the edible part, respectively) [27].

#### 3.2.4. Fatty Acids

The oil was extracted from *P. foetida* fruits using n-hexane in Soxhlet apparatus and with the Folch method with a mixture of chloroform/methanol. The yield (in percentage) of oil extraction with the Folch method was higher in nutmeg and anise extracted, compared to the Soxhlet extraction [29]. In terms of the fatty acid profile, the results in Table 3 show that the percentage of saturated fatty acids (myristic acid, palmitic acid, and stearic acid) was 25.82%, and the palmitic acid content (18.18%) was higher, comparatively. However, the percentage of unsaturated fatty acids (linoleic acid and oleic acid) was 74.18%. The most prevalent unsaturated fatty acid was linoleic acid (44.69%). The results indicated that *P. foetida* fruits have a high degree of unsaturated fatty acids, which is similar to those of *P. edulis Sims var. edulis*, *P. edulis Sims var. flavicarpa*, *Kawanda hybrid* and *P. maliformis* L. [30].

### 3.3. Functional Characterisation of the Powder

#### 3.3.1. TPC, TFC and Antioxidant Activity

As shown in Table 4, the TPC varied from 45.4 to 833 mg GAE/100 g in the lyophilized powder. Acidic NEP in the inedible portion had the highest TPC (833 mg GAE/100 g), followed by alkaline NEP in the inedible portion (622 mg GAE/100 g) and EP in the edible portion had the lowest TPC (45.4 mg GAE/100 g), which indicated that insoluble polyphenolic compounds were the representative form of phenols. The inedible portion sample presented a total phenolic level (1658.6 mg GAE/100 g), which was about two times higher than the edible portion sample (871.9 mg GAE/100 g). However, in other *Passiflora* fruits, such as *P. mollissima* (246 mg GAE/100 g of fresh inedible portion and 635 mg GAE/100 g of fresh edible portion, respectively) and *P. tarminiana* (288 mg GAE/100 g of fresh inedible portion and 1018 mg GAE/100 g of fresh edible portion, respectively), the content of phenolics in the edible portion was higher than that in the inedible portion [31]. The reason for the difference may be due genetic variation, environmental conditions, or extraction methods. 

The TFC ranged from 84 to 1163 mg RUE/100 g in the lyophilized powder. The alkaline NEP in the edible portion had the highest TFC (1163 mg RUE/100 g), followed by acidic NEP in the edible portion (814 mg RUE/100 g), and alkaline NEP in the inedible portion (84 mg RUE/100 g) had the lowest TFC. In addition, insoluble polyphenolic compounds were the main form of flavonoids in the edible portion, but soluble polyphenolic compounds were the main form of flavonoids in the inedible portion. The TFC of the edible portion powder (2216 mg RUE/100 g powder) was higher than that of the inedible portion (1350 mg RUE/100 g powder), which was similar to hamidazao (375.42 mg RUE/100 g of the edible portion powder and 276.43 mg RUE/100 g of inedible portion powder, respectively), but different from muzao (384 mg RUE/100 g of edible portion powder and 397.61 mg RUE/100 g of inedible portion powder, respectively) [32]. Moreover, the TPC was higher than the TFC in the inedible portion, which was similar to that of many fruits, such as strawberries (363.7 mg GAE/100 g FW and 14.6 mg RUE/100 g FW, respectively), oriental plums (668.0 mg GAE/100 g FW and 37.6 mg RUE/100 g FW, respectively), mulberries (1515.9 mg GAE/100 g FW and 250.1 mg RUE/100 g FW, respectively), and loquats (199.4 mg GAE/100 g FW and 14.2 mg RUE/100 g FW, respectively) [21]. However, the TFC was higher than the TPC in the edible portion, which demonstrated that flavonoids were the primary polyphenolic compounds in the edible portion. 

The DPPH and ABTS methods are widely used to evaluate the antioxidant activities of a food matrix, which is based on the reduction ability of a sample of antioxidants on the DPPH and ABTS through an electron transfer reaction [22]. Lower SC_50_ values indicate higher antioxidant activity. The SC_50_ values for the DPPH and ABTS radical scavenging activities of ethanolic extracts of all the extractions were presented in Table 4. All the extractions showed effects on the DPPH scavenging activities, with a range of SC_50_ values from 2.75 to 6.38 mg/mL; for the ABTS scavenging activities, SC_50_ ranged from 0.447 mg/mL to 9.8 mg/mL. 

The results of the DPPH study showed that the DPPH SC_50_ of alkaline NEP in the edible portion (2.75 mg/mL) was lower than that of EP (5.88 mg/mL) and acidic NEP (5.61 mg/mL). For the analysis of the inedible portion, the lowest DPPH SC_50_ was acidic NEP (5.26 mg/mL), followed by EP (6.3 mg/mL) and alkaline NEP (6.38 mg/mL). The results of ABTS analysis indicated that the lowest ABTS SC_50_ was alkaline NEP both in the edible portion and the inedible portion (0.447 mg/mL and 2.062 mg/mL, respectively), followed by EP (4.42mg/mL and 3.17 mg/mL, respectively) and acidic NEP (7.6 mg/mL and 9.8 mg/mL, respectively). Overall, the DPPH (2.75 mg/mL) and ABTS (0.447 mg/mL) radical scavenging activities of alkaline NEP in the edible portion showed higher antioxidant activities than other extractions, respectively, but it was a little lower than those of commercial natural and synthetic antioxidants used in the food industry, such as Trolox (SC_50_ = 0.74 mg/mL and 0.26 mg/mL, respectively). 

#### 3.3.2. Identification of Polyphenolics

**Figure 1:**
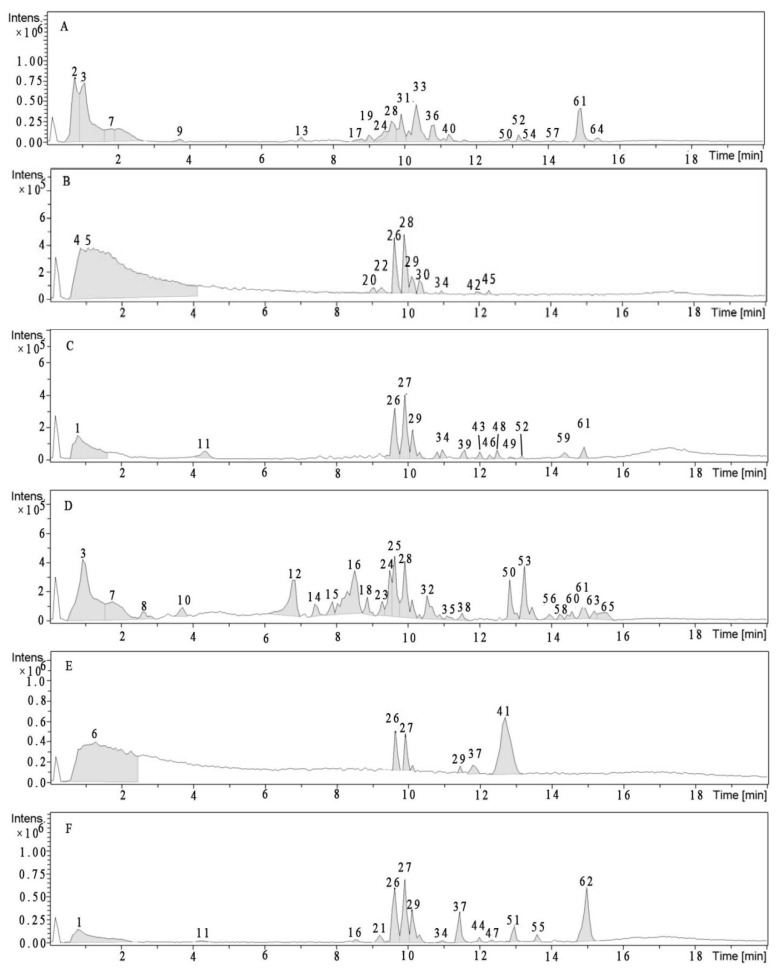
Representative total ion chromatogram (TIC) of (**A**) edible portion EP, (**B**) edible portion acidic NEP, (**C**) edible portion alkaline NEP, (**D**) inedible portion EP, (**E**) inedible portion acid NEP, and (**F**) inedible portion alkaline NEP in positive ion mode by UPLC-Q-TOF-MS^E^.

The UPLC-Q-TOF-MS^E^ analysis of the samples (Table 5 and Figure 1) allowed the tentative identification of 65 constituents, including 39 free phenolics, 14 insoluble glycoside phenolics and 22 insoluble ester phenolics. Most of the free phenolics and soluble phenolic esters in the edible and inedible parts were different, except compounds **3**, **24**, **28**, **50** and **61**. Two insoluble glycoside phenolics (compounds **26** and **29**) and six insoluble ester phenolics (compounds **1**, **11**, **26**, **27**, **29** and **34**) were discovered both in the edible part and inedible part. The compounds **26** and **29** existed in all acid NEP and alkaline NEP samples.

Caffeoylglucaric acid (compound **2**) (t_R_ = 0.81 min) with an [M − H]^−^ ion at *m*/*z* 371.0882 and a fragment ion at *m*/*z* 191.0144 was identified as caffeoylglucaric acid. Two other caffeic acid derivatives (compounds **8** and **12**) exhibited [M − H]^−^ ions at *m*/*z* 553.1413 and 625.1631, respectively, which were identical to the data reported in the literature [33].

Compound **20** (t_R_ = 9.06 min) with an [M − H]^−^ ion at *m*/*z* 285.0407, was identified as luteolin. Additionally, compound **36** (t_R_ = 11.25 min) with an [M − H]^−^ ion at *m*/*z* 343.0858 and a fragment ion at *m*/*z* 285.0410 was classified as a luteolin derivative [34]. 

Compounds **47** (*t*_R_ = 12.34 min) and **61** (*t*_R_ = 14.88 min), with fragment ions at *m*/*z* 163.0378, were identified as *p*-coumaric acid derivatives [35]. Moreover, compounds **30** (*t*_R_ = 10.31 min) and **60** (*t*_R_ = 14.57 min), with [M − H]^−^ ions at *m*/*z* 313.0734 and 341.2741, respectively, were identified as the esterified derivatives, pinobanksin-3-*O*-acetate and pinobanksin-3-*O*-butyrate [36]. The dimethoxylated flavonol peak (compound **34**) was equivalent to that of kaempferol and both compounds had an [M − H]^−^ at *m*/*z* 329.2362 [37]. 

Quinine dimer (compound **3**), with an [M − H]^−^ ion at *m*/*z* 383.0517, under fragmentation lost a quinic acid followed by another quinic acid bond [38]. Additionally, compound **4**, with an [M − H]^−^ ion at *m*/*z* 481.0236, showed identical fragmentation (*m*/*z* 191.0146) and was tentatively classified as quinic acid derivative [14]. Furthermore, compound **6** (*t*_R_ = 1.28 min), with an [M − H]^−^ ion at *m*/*z* 358.8792, was identified as rosmarinic acid [39]). In addition, compound **9** (*t*_R_ = 3.65 min), with an [M − H]^−^ ion at *m*/*z* 481.1134, was identified as silymarin [40].

Compound **11** (*t*_R_ = 14.7 min) was identified as hispidulin-7-*O*-hexoside through a comparison with data reported in the literature [41]. According to their fragmentation patterns and through comparisons with data reported in literature [33], compounds **14**, **15, 35**, **47**, **56** and **63** with [M − H]^−^ ions at *m*/*z* 593.1434, 417.1582, 385.2271, 433.2594, 529.2990 and 487.3090, were assigned as apigenin-6, 8-di-C-glycoside, coumarylquinic acid derivative, feruloylglucaric acid, *p*-coumaric acid, 1-*O*-caffeoyl-5-*O*-feruloylquinic acid and caffeic acid-*O*-hexoside-*O*-rhamnoside, respectively. Compound **13** (t_R_ = 11.9 min), with [M − H]^−^ ions at *m*/*z* 755.1698, was identified as 3-*O*-rhamnosyl-glucoside 7-*O*-rhamnoside through a comparison with data reported in the literature [42]. Compound **59** (*t*_R_ = 14.36 min), with [M − H]^−^ at *m*/*z* 623.2774, was identified as isorhamnetin 3-*O*-glucoside 7-*O*-rhamnoside through a comparison with data reported in the literature [42]. 

Compounds **32**, **42** and **62**, with [M − H]^−^ ions at *m*/*z* 447.2273, 593.1262 and 607.4342, were assigned as C-glycosyl flavonoids vicenin-2, isoorientin and spinosyn, respectively [34].

The identification of compounds **1**, **5**, **7**, **16**, **18**, **21**–**23**, **25**–**29**, **31**, **33**, **37**–**41**, **43**–**46**, **48**–**50**, **52**–**54**, **57**, **58**, **64** and **65** could not be established because their MS^n^ data that did not provide enough information concerning their chemical structures, which suggested that these molecules might be new compounds. Particularly, compounds **24** and **28** exhibited [M − H]^−^ ions at *m*/*z* 723.4510 and 836.5284, respectively, both showing a sequential loss of formate adduct [M–H–HCOOH]^−^ , which was a common phenomenon of the passionfruit [33]. Dimers (compounds **7**, **31** and **33**), with an [M − H]^−^ ion at *m*/*z* 781.1470, 627.1411 and 659.1250, were under fragments 439.0800, 313.0737 and 329.0690, respectively. The base peaks of **26**, **27** and **29** were generated with [M − H]^−^ ions at *m*/*z* 713.4510, 826.5030 and 939.5540, respectively, both showing sequential loss of a chlorine adduct [M − H − Cl]^−^. Based on the MS/MS^E^ data, compounds **24** and **39** were characterised as one set of isomers, and **43** and **46** as another set. These compounds still need further investigation for the identification of their structures. 

## 4. Conclusions

In this study, a detailed chemical composition of wild bacca fruit of *P. foetida* was determined for the first time. The results showed that the fruits could be considered as a new natural source of health-promoting compounds, including essential amino acids, unsaturated fatty acids, minerals and antioxidant phenolic compounds. Taken together, the compositional data on sim fruit obtained in our study indicated that the fruits had an unusual nutritional composition that could gain further attention from nutritionists, health specialists, as well as the food industry, and, in turn, promote the use of this neglected and underutilized fruits in the future.

## Figures and Tables

**Table 1:** Weight, size, chromatic coordinates, hue and chroma of *P. foetida* fruits.

Weight (g)	Fruit Shape Index			Color Parameters		
	Length (cm)	Diameter (cm)	L/D ^a^	L *	a *	b *
3.3 ± 0.2 (*n* = 25)	1.62 ± 0.13 (*n* = 25)	1.88 ± 0.13 (*n* = 25)	0.86 ± 0.04 (*n* = 25)	53 ± 1 (*n* = 6)	17 ± 2 (*n* = 6)	87 ± 2 (*n* = 6)

Data correspond to the mean ± SD. ^a^ L/D refer to the length/diameter. L * = lightness, a * = red-green hue, b * = yellow-blue hue.

**Table 2:** Proximate compositions of *P. foetida* fruits.

Compositions (Unit)	Data
Edible portion (g/100 g fresh fruit)	62.73–89.85
Inedible portion (g/100 g fresh fruit)	10.15–37.27
Water (g/100 g fresh fruit)	76 ± 1
Total fat (g/100 g fresh fruit)	13 ± 2
Total Protein (g/100 g fresh fruit)	0.15 ± 0.01
Ash (g/100 g fresh fruit)	1.7 ± 0.3
Total sugars (g/100 g fresh fruit)	3.6 ± 0.1
pH	4.5 ± 0.1
Total Energy (kJ)	991 ± 88

**Table 3:** Nutritional composition (fatty acids, amino acids, minerals, sugars, and organic acids) of the fruit, expressed on a fresh weight (FW) basis and per 100 g edible portion.

**Sugars (g)**	**Per 100 g of FW**	**Percentage of Total Sugars (%)**
Glucose	1.6 ± 0.1	47.90
Fructose	1.6 ± 0.1	47.90
Sucrose	0.14 ± 0.01	4.20
Total	3.34	
**Organic Acids (mg)**	**Per 100 g of FW**	**Percentage of Total Organic Acids (%)**
Oxalic acid	0.07 ± 0.01	29.17
Tartaric acid	0.01 ± 0.00	4.17
Malic acid	0.01 ± 0.00	4.17
Ascorbic acid	0.03 ± 0.00	12.50
Citric acid	0.12 ± 0.01	50.00
Total	0.24	
**Amino Acids (mg)**	**Per 100 g of FW**	**Percentage of Total Amino Acids (%)**
Aspartic acid	110 ± 3	10.02
Serine	57 ± 2	5.20
Glutamic	203 ± 7	18.51
Glycine	63 ± 1	5.74
Alanine	58.2 ± 0.6	5.31
Cystine	18 ± 6	1.64
Tyrosine	17 ± 3	1.55
Phenylalanine	77 ± 4	7.02
Arginine	140 ± 4	12.76
Proline	46 ± 3	4.20
Methionine	10 ± 2	0.91
Threonine	40.7 ± 0.8	3.71
Isoleucine	40.4 ± 0.3	3.68
Leucine	74 ± 2	6.75
Lysine	58.6 ± 0.4	5.34
Histidine	28.6 ± 0.3	2.61
Valine	55.2 ± 0.7	5.03
Cystine + Methionine	28 ± 8	2.55
Tyrosine + Phenylalanine	118 ± 5	10.76
EAA	286 ± 1	26.07
NEAA	811 ± 133	73.93
Total	1097	
**Minerals (mg)**	**Per 100 g of FW**	**Percentage of Total Minerals (%)**
Manganese	0.32 ± 0.03	0.05
Copper	0.21 ± 0.02	0.04
Iron	0.89 ± 0.07	0.15
Zinc	1.03 ± 0.09	0.17
Phosphorus	86 ±7	14.44
Sodium	10± 2	1.68
Magnesium	40 ± 2	6.71
Potassium	451 ± 7	75.70
Calcium	6.3 ± 0.4	1.06
Total	595.75	
**Fatty Acids**		**Percentage of Total Fatty Acids (%)**
Myristic acid (C14:0)		0.48
Palmitic acid (C16:0)		18.18
Linoleic acid (C18:2, C9C12)		44.69
Oleic acid (C18:1, C9)		29.49
Stearic acid (C18:0)		7.16

**Table 4:** Total phenolic contents (TPC), total flavonoid contents (TFC) and radical scavenging capacity of powder of *P. foetida* fruits.

*P. foetida* Powder		Polyphenolic Compounds mg/100g Powder			SC_50_ (mg/mL) ^g^	
		TPC	TFC	Total	DPPH	ABTS
Edible portion	EP	45.4 ± 0.1 ^f^	239 ± 1 ^d^	284.4	5.88 ± 0.09 ^cd^	4.42 ± 0.04 ^d^
	Acid NEP	608 ± 5 ^c^	814 ± 24 ^b^	1422	5.61 ± 0.07 ^bc^	7.6 ± 0.1 ^f^
	Alkaline NEP	218.5 ± 0.3 ^d^	1163 ± 2 ^a^	1381.5	2.75 ± 0.04 ^a^	0.447 ± 0.001 ^a^
	Total	871.9	2216	3087.6		
Inedible portion	EP	203.6 ± 0.9 ^e^	753 ± 7 ^b^	956.6	6.3 ± 0.5 ^d^	3.17 ± 0.09 ^c^
	Acid NEP	833 ± 5 ^a^	513 ± 61 ^c^	1346	5.26 ± 0.03 ^b^	9.8 ± 0.1 ^e^
	Alkaline NEP	622 ± 2 ^b^	84 ± 15 ^e^	706	6.38 ± 0.08 ^d^	2.062 ± 0.007 ^b^
	Total	1658.6	1350	3008.6		
Reference compound		Gallic acid	Rutin		Trolox	Trolox

Data represent the mean values for each sample ± standard deviations (*n* = 3). Different letters (a, b, c, d, e, f) within columns denote statistically significant differences (*p* < 0.05) according to Tukey test. ^g^ SC_50_, concentration of the sample that decreases the initial 1,1-diphenyl-2-picryl-hydrazl (DPPH) and 2,2-azinobis-(3-ethylbenzthiazoline-6-sulfonic acid) (ABTS) absorbance. EP, extractable phenolics; NEP, non-extractable phenolic.

**Table 5:** Characterisation of phenolic compounds of *P. foetida* fruits by UPLC-Q-TOF-MS^E^.

Compound	T_R_ (min)	(ESI)^−^ (*m*/*z* Abundance)	Proposed Structure	Edible Portion			Inedible Portion		
				EP	Acid NEP	Alkaline NEP	EP	Acid NEP	Alkaline NEP
**1**	0.79	MS: 794.4155; MS/MS: 268.8039	Unknown			+			+
**2**	0.81	MS: 371.0882; MS/MS: 191.0144	Caffeoylglucaric acid	+					
**3**	0.96	MS: 383.0517; MS/MS: 191.0162	Quinine dimer	+			+		
**4**	1.03	MS: 481.0236; MS/MS: 191.0146	Quinic acid derivative						
**5**	1.06	MS: 208.9383; MS/MS: 96.9636	Unknown		+				
**6**	1.28	MS: 358.8792; MS/MS: 264.9029	Rosmarinic acid					+	
**7**	1.75	MS: 781.1470; MS/MS: 439.0800	Unknown	+			+		
**8**	2.60	MS: 553.1413; MS/MS: 455.1866, 167.0313	Caffeic acid derivative				+		
**9**	3.65	MS: 481.1134; MS/MS: 473.0759, 137.0221	Silymarin	+					
**10**	3.71	MS:445.1406; MS/MS: 443.1954, 137.0217	Lucuminic acid				+		
**11**	4.34	MS: 703.1230; MS/MS: 461.0935, 219.0506	Hispidulin-7-*O*-hexoside derivative			+			+
**12**	6.80	MS: 625.1631; MS/MS: 301.0937	Caffeic acid derivative				+		
**13**	7.12	MS: 755.1698; MS/MS: 319.0830	Quercetin 3-*O*-rhamnosyl-glucoside 7-*O*-rhamnoside	+					
**14**	7.40	MS: 593.1434; MS/MS: 473.1109, 353.0709	Apigenin-6,8-di-C-glycoside (vicenin-2)				+		+
**15**	7.89	MS: 417.1582; MS/MS: 206.0808	Coumarylquinic acid derivative				+		
**16**	8.51	MS: 629.1147; MS/MS: 675.2466, 319.1335	Unknown				+		
**17**	8.79	MS: 497.0879; MS/MS: 299.0581, 284.0329	Chrysoeriol derivative	+					
**18**	8.86	MS: 671.2571; MS/MS: 249.1339	Unknown				+		
**19**	9.02	MS: 600.3834; MS/MS: 285.0406	Luteolin derivative	+					
**20**	9.06	MS: 285.0407; MS/MS: 149.0193	Luteolin		+				
**21**	9.21	MS: 600.3899; MS/MS: 564.4132, 289.1176	Unknown						+
**22**	9.27	MS: 676.3797; MS/MS: 346.1848, 110.9766	Unknown		+				
**23**	9.28	MS: 307.0856; MS/MS: 161.0402	Unknown				+		
**24**	9.42	MS: 723.4727; MS/MS: 677.4750, 329.0685	luteolin-3′-*O*-dirhamnoside-7-*O*-rhamnoside	+			+		
**25**	9.59	MS: 790.5349; MS/MS: 707.2673	Unknown				+		
**26**	9.62	MS:713.4510; MS/MS: 677.4817	Unknown		+	+		+	+
**27**	9.66	MS: 826.5030; MS/MS: 790.5349, 329.0708	Unknown			+		+	+
**28**	9.90	MS: 836.5284; MS/MS: 790.5349	Unknown	+	+		+		
**29**	10.11	MS: 939.5540; MS/MS: 903.5825	Unknown		+	+		+	+
**30**	10.31	MS: 313.0734; MS/MS: 283.0245	Pinobanksin-3-*O*-acetate		+				
**31**	10.32	MS: 627.1411; MS/MS: 313.0737	Unknown	+					
**32**	10.56	MS: 447.2273; MS/MS: 191.0530	Vicenin-2				+		
**33**	10.78	MS: 659.1250; MS/MS: 329.0690	Unknown	+					
**34**	10.94	MS: 329.2362; MS/MS: 201.1069	Kaempferol-methoxy-methyl ether		+	+			+
**35**	11.09	MS: 385.2271; MS/MS: 267.1972	Feruloylglucaric acid				+		
**36**	11.25	MS: 343.0858; MS/MS: 313.0373, 285.0410	Luteolin derivative	+					
**37**	11.43	MS: 323.2037; MS/MS: 287.2262	Unknown					+	+
**38**	11.50	MS: 311.1892; MS/MS: 267.1970	Unknown				+		
**39**	11.58	MS: 723.4726; MS/MS: 379.2249	Unknown			+			
**40**	11.64	MS: 379.0651; MS/MS: 328.0612, 313.0413	Unknown	+					
**41**	11.78	MS: 501.1454; MS/MS: 389.1616, 367.1838	Unknown					+	
**42**	11.91	MS: 593.1262; MS/MS: 417.1005, 209.0401	Isoorientin		+				
**43**	11.99	MS: 653.2940; MS/MS: 653.2910	Unknown			+			
**44**	11.99	MS: 639.4592; MS/MS: 337.2124	Unknown						+
**45**	12.26	MS: 540.2528; MS/MS: 425.2245, 110.9778	Unknown		+				
**46**	12.26	MS: 653.2940; MS/MS: 653.2910	Unknown			+			
**47**	12.34	MS: 433.2594; MS/MS: 287.2228, 163.0378	*p*-coumaric acid derivative						+
**48**	12.48	MS: 763.4315; MS/MS: 653.2859, 399.1997	Unknown			+			
**49**	12.83	MS: 1187.2153; MS/MS: 739.4765, 324.7376	Unknown			+			
**50**	12.83	MS: 721.3361; MS/MS: 397.1392, 277.2188	Unknown	+			+		
**51**	12.95	MS: 447.2760; MS/MS: 415.2473	Luteolin 8-C-hexoside (orientin)						+
**52**	13.17	MS: 297.2446; MS/MS: 183.1340	Unknown	+		+			
**53**	13.24	MS: 549.2817; MS/MS: 277.2167	Unknown				+		
**54**	13.38	MS: 474.2656; MS/MS: 277.2171	Unknown	+					
**55**	13.60	MS: 593.4174; MS/MS: 557.4400, 287.2226	Kaempferol-3-*O*-rutinoside						+
**56**	13.94	MS: 529.2990; MS/MS: 279.2320	1-*O*-Caffeoyl-5-*O*-feruloylquinic acid				+		
**57**	14.15	MS: 476.2824; MS/MS: 279.2334	Unknown	+					
**58**	14.22	MS: 385.3012; MS/MS: 325.1847, 263.2395	Unknown				+		
**59**	14.36	MS: 623.2774; MS/MS: 461.2278, 446.2038	Isorhamnetin 3-*O*-glucoside 7-*O*-rhamnoside			+			
**60**	14.57	MS: 341.2741; MS/MS: 339.2020	Pinobanksin-3-*O*-butyrate				+		
**61**	14.88	MS: 339.2374; MS/MS: 163.1074	*p*-coumaric acid derivative	+		+	+		
**62**	14.97	MS: 607.4342; MS/MS: 607.4342	Spinosin						+
**63**	15.19	MS: 487.3090; MS/MS: 341.3043	Caffeic acid-*O*-hexoside-*O*-rhamnoside				+		
**64**	15.40	MS: 743.4882; MS/MS: 739.4757, 389.1516, 268.7993	Unknown	+					
**65**	15.47	MS: 671.2448; MS/MS: 277.2184	Unknown				+		

+: The compound was detected in the extract(s).
